# Supplementary material for: Multimodal Music Perception Engages Motor Prediction: A TMS Study
Source: Front Neurosci. 2018 Oct 18;12:736. doi: 10.3389/fnins.2018.00736 (PMC6201045; doi:10.3389/fnins.2018.00736)
Supplement: Supplementary file 1 [file Data_Sheet_1.PDF]

Table S1

FDI Post-hoc Multiple Comparisons of Conditions

	Difference	Lower CI bound	Upper CI bound	p-adjusted	
AV-lag - A	0.17061382	0.03874108	0.30248657	0.00515512	**
AV - A	0.08422372	-0.047649	0.21609646	0.3522313	
V - A	0.0355776	-0.0962951	0.16745035	0.89816635	
AV - AV-lag	-0.0863901	-0.2182628	0.04548264	0.32940244	
V - AV-lag	-0.1350362	-0.266909	-0.0031635	0.0424534	*
V - AV	-0.0486461	-0.1805189	0.08322663	0.7760927	

Table S2

FDI Post-hoc Multiple Comparisons of Interactions

	Difference	Lower CI bound	Upper CI bound	p-adjusted	
FDI:A - FDM:A	-0.1475006	-0.3678552	0.07285399	0.45445774	
FDM:AV-lag - FDM:A	-0.0235307	-0.2438852	0.19682394	0.99998084	
FDI:AV-lag - FDM:A	0.2172577	-0.0030969	0.43761229	0.05645305	.
FDM:AV - FDM:A	-0.0211121	-0.2414667	0.19924247	0.99999089	
FDI:AV - FDM:A	0.04205896	-0.1782956	0.26241355	0.99905906	
FDM:V - FDM:A	-0.0420251	-0.2623797	0.17832945	0.99906397	
FDI:V - FDM:A	-0.0343202	-0.2546748	0.18603434	0.99975372	
FDM:AV-lag - FDI:A	0.12396995	-0.0963846	0.34432454	0.67588841	
FDI:AV-lag - FDI:A	0.3647583	0.14440371	0.58511289	2.08E-05	**
FDM:AV - FDI:A	0.12638848	-0.0939661	0.34674307	0.6536736	
FDI:AV - FDI:A	0.18955956	-0.030795	0.40991415	0.15074547	
FDM:V - FDI:A	0.10547546	-0.1148791	0.32583005	0.82728562	
FDI:V - FDI:A	0.11318035	-0.1071742	0.33353494	0.76904611	
FDI:AV-lag - FDM:AV-lag	0.24078835	0.02043376	0.46114294	0.02125037	*
FDM:AV - FDM:AV-lag	0.00241853	-0.2179361	0.22277313	1	
FDI:AV - FDM:AV-lag	0.06558961	-0.154765	0.2859442	0.98509273	
FDM:V - FDM:AV-lag	-0.0184945	-0.2388491	0.2018601	0.99999634	
FDI:V - FDM:AV-lag	-0.0107896	-0.2311442	0.20956499	0.99999991	
FDM:AV - FDI:AV-lag	-0.2383698	-0.4587244	-0.0180152	0.02362913	*
FDI:AV - FDI:AV-lag	-0.1751987	-0.3955533	0.04515585	0.23215028	
FDM:V - FDI:AV-lag	-0.2592828	-0.4796374	-0.0389283	0.00906161	**
FDI:V - FDI:AV-lag	-0.2515779	-0.4719325	-0.0312234	0.01303703	*
FDI:AV - FDM:AV	0.06317107	-0.1571835	0.28352567	0.98804767	
FDM:V - FDM:AV	-0.020913	-0.2412676	0.19944157	0.99999147	
FDI:V - FDM:AV	-0.0132081	-0.2335627	0.20714646	0.99999965	
FDM:V - FDI:AV	-0.0840841	-0.3044387	0.13627049	0.94115843	
FDI:V - FDI:AV	-0.0763792	-0.2967338	0.14397539	0.96470741	
FDI:V - FDM:V	0.0077049	-0.2126497	0.22805949	0.99999999	

Table S3

FDM Post-hoc Multiple Comparisons of Conditions

	Difference	Lower CI bound	Upper CI bound	p-adjusted	
AV-lag - A	0.06722797	-0.0931759	0.22763185	0.69997393	
AV - A	-0.0187198	-0.1791237	0.14168407	0.99044555	
V - A	-0.0919055	-0.2523094	0.06849835	0.450331	
AV - AV-lag	-0.0859478	-0.2463517	0.0744561	0.50977878	
V - AV-lag	-0.1591335	-0.3195374	0.00127039	0.05272527	.
V - AV	-0.0731857	-0.2335896	0.08721817	0.64037109	

Table S4

FDM Post-hoc Multiple Comparisons of Interactions

	Difference	Lower CI bound	Upper CI bound	p-adjusted
FDM:A - FDI:A	0.08583725	-0.1822272	0.3539017	0.97718666
FDI:AV-lag - FDI:A	0.05944625	-0.2086182	0.32751071	0.99751806
FDM:AV-lag - FDI:A	0.16084692	-0.1072175	0.42891138	0.59819191
FDI:AV - FDI:A	-0.0337564	-0.3018209	0.23430802	0.99994068
FDM:AV - FDI:A	0.08215404	-0.1859104	0.3502185	0.9822483
FDI:V - FDI:A	-0.0797978	-0.3478623	0.18826663	0.98500718
FDM:V - FDI:A	-0.018176	-0.2862404	0.24988846	0.99999916
FDI:AV-lag - FDM:A	-0.026391	-0.2944554	0.24167346	0.99998895
FDM:AV-lag - FDM:A	0.07500968	-0.1930548	0.34307413	0.98959417
FDI:AV - FDM:A	-0.1195937	-0.3876581	0.14847077	0.87319152
FDM:AV - FDM:A	-0.0036832	-0.2717477	0.26438125	1
FDI:V - FDM:A	-0.1656351	-0.4336995	0.10242939	0.56072836
FDM:V - FDM:A	-0.1040132	-0.3720777	0.16405122	0.93561165
FDM:AV-lag - FDI:AV-lag	0.10140067	-0.1666638	0.36946512	0.94351906
FDI:AV - FDI:AV-lag	-0.0932027	-0.3612671	0.17486177	0.96394287
FDM:AV - FDI:AV-lag	0.02270779	-0.2453567	0.29077225	0.99999607
FDI:V - FDI:AV-lag	-0.1392441	-0.4073085	0.12882038	0.75797241
FDM:V - FDI:AV-lag	-0.0776222	-0.3456867	0.19044221	0.98725155
FDI:AV - FDM:AV-lag	-0.1946034	-0.4626678	0.0734611	0.34450424
FDM:AV - FDM:AV-lag	-0.0786929	-0.3467573	0.18937158	0.98618196
FDI:V - FDM:AV-lag	-0.2406447	-0.5087092	0.02741971	0.11463664
FDM:V - FDM:AV-lag	-0.1790229	-0.4470874	0.08904154	0.45691917
FDM:AV - FDI:AV	0.11591048	-0.152154	0.38397493	0.89035399
FDI:V - FDI:AV	-0.0460414	-0.3141058	0.22202307	0.99952575
FDM:V - FDI:AV	0.01558044	-0.252484	0.2836449	0.99999971
FDI:V - FDM:AV	-0.1619519	-0.4300163	0.10611259	0.58956522
FDM:V - FDM:AV	-0.10033	-0.3683945	0.16773442	0.94655766
FDM:V - FDI:V	0.06162183	-0.2064426	0.32968629	0.99688587
